# Effects of altered *α*- and *β*-branch carotenoid biosynthesis on photoprotection and whole-plant acclimation of *Arabidopsis* to photo-oxidative stress

**DOI:** 10.1111/j.1365-3040.2012.02586.x

**Published:** 2013-02

**Authors:** ROSANNA CALIANDRO, KERSTIN A NAGEL, BERND KASTENHOLZ, ROBERTO BASSI, ZHIRONG LI, KRISHNA K NIYOGI, BARRY J POGSON, ULRICH SCHURR, SHIZUE MATSUBARA

**Affiliations:** 1 IBG-2: Pflanzenwissenschaften, Forschungszentrum Jülich52425 Jülich, Germany; 2 Dipartimento di Biotecnologie, Università degli Studi di Verona37134 Verona, Italy; 3 Department of Plant and Microbial Biology, Howard Hughes Medical InstituteUniversity of California; 4 Physical Biosciences Division, Lawrence Berkeley National LaboratoryBerkeley, CA 94720-3102, USA; 5 Australian Research Council Centre of Excellence in Plant Energy Biology, Research School of Biology, Australian National UniversityCanberra, ACT 0200, Australia

**Keywords:** *Arabidopsis thaliana*, carotene, leaf growth, lutein, non-photochemical quenching, root growth, sunflecks, xanthophyll cycle

## Abstract

Functions of *α*- and *β*-branch carotenoids in whole-plant acclimation to photo-oxidative stress were studied in *Arabidopsis thaliana* wild-type (wt) and carotenoid mutants, *lutein deficient* (*lut2*, *lut5*), *non-photochemical quenching1* (*npq1*) and *suppressor of zeaxanthin-less1* (*szl1*) *npq1* double mutant. Photo-oxidative stress was applied by exposing plants to sunflecks. The sunflecks caused reduction of chlorophyll content in all plants, but more severely in those having high *α*- to *β*-branch carotenoid composition (*α*/*β*-ratio) (*lut5*, *szl1npq1*). While this did not alter carotenoid composition in wt or *lut2*, which accumulates only *β*-branch carotenoids, increased xanthophyll levels were found in the mutants with high *α*/*β*-ratios (*lut5*, *szl1npq1*) or without xanthophyll-cycle operation (*npq1*, *szl1npq1*). The PsbS protein content increased in all sunfleck plants but *lut2*. These changes were accompanied by no change (*npq1*, *szl1npq1*) or enhanced capacity (wt, *lut5*) of NPQ. Leaf mass per area increased in *lut2*, but decreased in wt and *lut5* that showed increased NPQ. The sunflecks decelerated primary root growth in wt and *npq1* having normal *α*/*β*-ratios, but suppressed lateral root formation in *lut5* and *szl1npq1* having high *α*/*β*-ratios. The results highlight the importance of proper regulation of the *α*- and *β*-branch carotenoid pathways for whole-plant acclimation, not only leaf photoprotection, under photo-oxidative stress.

## INTRODUCTION

Carotenoids play important roles in photosynthetic membranes (thylakoids), such as folding and stabilization of pigment-binding proteins, regulation of light harvesting and photoprotection (Yamamoto & Bassi 1996). Typically, leaves accumulate *β*-carotene (*β*-C), lutein (L), violaxanthin (V) and neoxanthin (N). In addition, part of V is converted to antheraxanthin (A) and zeaxanthin (Z) by the enzyme V de-epoxidase, which is activated in excess light conditions. Upon return to low light (LL), that is, without concurrent activity of V de-epoxidase, epoxidation by Z epoxidase restores the V pool in the so-called ‘xanthophyll cycle’ (or V cycle). The Z enhances scavenging of reactive oxygen species (ROS) and thermal energy dissipation [commonly measured as non-photochemical quenching (NPQ) of fluorescence] in the thylakoids, thus playing central roles in protection against photo-oxidative damage (Havaux & Niyogi 1999; Müller, Li & Niyogi 2001; Havaux, Dall’Osto & Bassi 2007).

The leaf carotenoid composition has been conserved throughout evolution of higher plants, which suggests distinct functions for each carotenoid species. Interestingly, two additional carotenoids, *α*-carotene (*α*-C) and L epoxide (Lx), can accumulate in large amounts in leaves of certain taxa, especially in shade environments (García-Plazaola, Matsubara & Osmond 2007; Matsubara *et al*. 2009) where they replace, respectively, *β-*C and L or the V-cycle pigments (V, A and Z) in pigment-protein complexes of photosystem II (PSII) and photosystem I (PSI) (Matsubara *et al*. 2007). Based on its predominant occurrence in shade leaves (Krause *et al*. 2001; Matsubara *et al*. 2009), an adaptive advantage of having *α*-C under LL conditions has been proposed. Accumulation of Lx can result in operation of a second light-dependent xanthophyll cycle between Lx and L (Lx cycle), which presumably is catalyzed by the enzymes V de-epoxidase and Z (or L) epoxidase (García-Plazaola *et al*. 2007). Similar to V-to-Z de-epoxidation, Lx-to-L de-epoxidation results in faster induction of NPQ upon illumination, suggesting involvement of both Z and L in regulation of light harvesting and NPQ (Matsubara *et al*. 2008, 2011; Esteban *et al*. 2010; Förster, Pogson & Osmond 2011).

**Figure 1:**
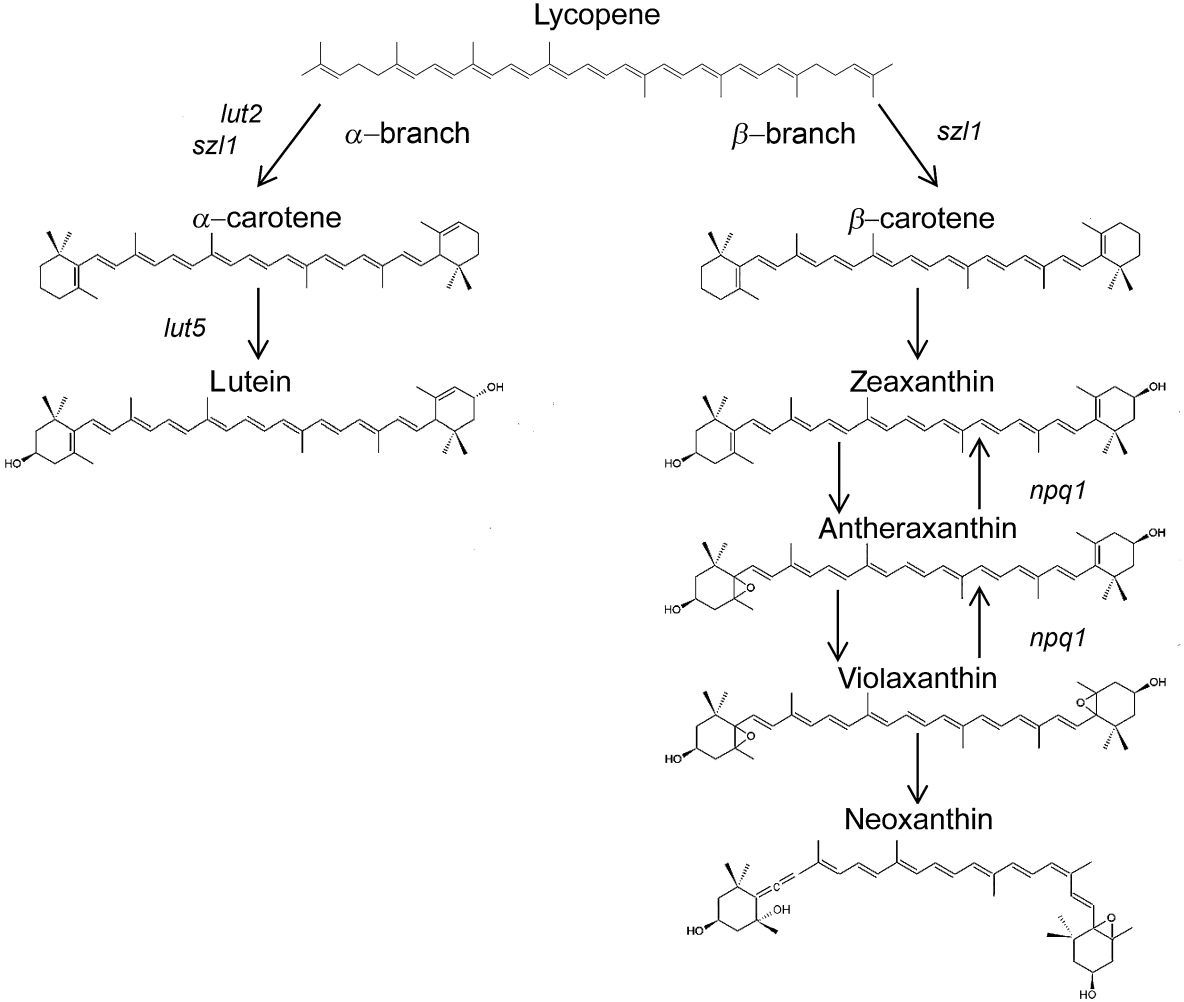
Two branches of the carotenoid biosynthetic pathway in higher plants. The enzyme activities impaired in *lut2*, *lut5*, *szl1npq1* and *npq1* mutants are also indicated.

Carotenoids are synthesized in the carotenoid biosynthetic pathway (DellaPenna & Pogson 2006) which is split into a *β*-branch (with two *β*-ionone rings) and an *α*-branch (with a *β*-ionone and an *ε*-ionone ring) by the activities of lycopene *β*- and *ε*-cyclase (Fig. 1). The first product of the *β*-branch, *β*-C, binds to the photosynthetic core complexes. The *β*-ring hydroxylation of *β*-C produces Z, which is followed by its epoxidation to A and V by Z epoxidase and further modification to N. In the *α*-branch, *ε*-cyclase and *ε*-hydroxylases are also needed to produce L, the most abundant carotenoid in photosynthetic tissues. In many plants *α*-C and Lx, the precursor and the epoxidation product of L, respectively, do not accumulate in leaves in significant amounts. In species in which these additional *α*-branch carotenoids occur, their pronounced accumulation in shade leaves leads to a high *α*-branch to *β*-branch carotenoid composition (*α*/*β*-ratio) that is reversed in sun leaves (Matsubara *et al*. 2009). The *β*-branch xanthophylls are essential for photoprotection (Dall’Osto *et al*. 2007b), but they cannot fully functionally replace the *α*-branch xanthophyll L, as shown in mutants of *Arabidopsis thaliana* (L.) *Heynh*. with altered carotenoid compositions (Dall’Osto *et al*. 2007b; Kalituho, Rech & Jahns 2007).

Compared with the increasing knowledge about the roles of different carotenoids in photoprotection revealed by studies using *Arabidopsis* carotenoid mutants and combining genetics, biochemistry, biophysics and physiology (e.g. Niyogi, Grossman & Björkman 1998; Havaux & Niyogi 1999; Dall’Osto *et al*. 2007b; Havaux *et al*. 2007; Johnson *et al*. 2007, 2009; Kalituho *et al*. 2007; Li *et al*. 2009; Cazzaniga *et al*. 2012), little attention has been paid to the importance of carotenoid biosynthesis in whole-plant acclimation to photo-oxidative stress. Leaf acclimation to irradiance involves marked modifications of leaf biochemistry as well as leaf thickness, structure and morphology (Evans & Poorter 2001; Terashima *et al*. 2006). Plant stress acclimation can also entail resource allocation between different organs or between young and old tissues of the same organ. A good example for such acclimation is enhanced (or less impaired) growth of roots compared with leaves in the face of nutrient limitation, resulting in altered root-shoot ratios (Poorter *et al*. 2012), or the well-known symptom of shade avoidance manifested by strong elongation of stems and petioles (Franklin 2008). Because changes in carotenoid composition are a hallmark of leaf photoacclimation, and furthermore, the *β*-branch pathway is upstream of the biosynthesis of abscisic acid (ABA) and strigolactones (SL), two important plant hormones that regulate shoot and root growth (Koornneef, Bentsink & Hilhorst 2002; Horvath *et al*. 2003; Kozuka *et al*. 2005; De Smet *et al*. 2006; Gomez-Roldan *et al*. 2008; Umehara *et al*. 2008; Koltai 2011), the carotenoid biosynthesis may play a key role in plant acclimation to light stress on molecular as well as leaf and whole-plant levels.

Thus, in order to study the impact of altered *α*- and *β*-branch carotenoid composition and biosynthesis on photoprotection and whole-plant stress acclimation, we examined the responses of four different *Arabidopsis* carotenoid mutants, *lut2*, *lut5*, *npq1* and *szl1npq1* (all in the Columbia-0 background), to photo-oxidative stress. The *lut2* lacks *ε*-cyclase (Fig. 1), which blocks the *α*-branch pathway and results in strong accumulation of the V-cycle pigments replacing L (Pogson *et al*. 1996). The *lut5* does not have a cytochrome P450 that catalyzes *β*-ring hydroxylation of *α*-C, leading to pronounced accumulation of *α*-C at the expense of *β*-C and the V-cycle pigments (Fiore *et al*. 2006; Kim & DellaPenna 2006). The *npq1* is deficient in V de-epoxidase and thus cannot employ Z-dependent photoprotective mechanisms under high-light (HL) stress (Niyogi *et al*. 1998). The *szl1npq1* is a double mutant which, besides lacking Z, has reduced amounts of *β*-branch carotenoids and remarkably high levels of L and *α*-C due to a point mutation in *β*-cyclase (Li *et al*. 2009). The pronounced accumulation of L in *szl1npq1* partly rescues the NPQ deficiency phenotype of *npq1*, confirming the ability of L to compensate, at least in part, for the function of Z in regulating light harvesting and NPQ (Li *et al*. 2009). It is important to note that none of these four *Arabidopsis* carotenoid mutants shows an altered or visibly impaired growth phenotype under non-stressful or LL conditions, indicating that their hormone synthesis is not strongly affected under these conditions. This allows us to investigate roles of carotenoids and their biosynthesis in stress-induced acclimatory responses.

Photo-oxidative stress was applied by exposing the LL-grown *Arabidopsis* plants (60 *µ*mol photons m^−2^ s^−1^) to repetitive short ‘sunflecks’ (1000 *µ*mol photons m^−2^ s^−1^, lasting ca. 20 s) every 6 min under the background illumination of 60 *µ*mol photons m^−2^ s^−1^ during the daytime. In highly variable natural light environments, sunflecks can expose shade-acclimated leaves to sudden and strong increase in irradiance. While they provide the major fraction of light energy available in shade environments and may thus improve carbon gain in some plants (Pearcy 1990), they can also be a source of photo-oxidative stress due to temporal excess of light (Logan *et al*. 1997; Watling *et al*. 1997; Adams *et al*. 1999). In *Arabidopsis*, short and strong sunflecks primarily induce photoprotective acclimation, such as decrease in leaf Chl content, up-regulation of NPQ, increase in the pool size of the V-cycle pigments and enhanced activity of superoxide dismutase (Alter *et al*. 2012). Hence, sunfleck treatments can be used to study photo-oxidative stress in *Arabidopsis* plants without concomitant up-regulation of photosynthesis, carbon gain and growth as is often found in leaves and plants under continuous HL.

The roles of *α*- and *β*-branch carotenoids in photoprotective acclimation were studied by monitoring changes in pigment composition, PSII efficiency and NPQ in fully expanded, mature leaves of *Arabidopsis* wild-type (wt) Columbia-0 and the four carotenoid mutants during the sunfleck treatment over 7 d. The impact of altered *α*- and *β*-branch carotenoid biosynthesis on stress responses was also evaluated from a whole-plant perspective by analysing leaf and root growth as well as seed production under the same conditions.

## MATERIALS AND METHODS

### Plant material and growth conditions

The four mutants of *A. thaliana* (L.) *Heynh*. used in this study are all in the Columbia-0 background: *lut2* (Pogson *et al*. 1996), *lut5* (Fiore *et al*. 2006; Kim & DellaPenna 2006), *npq1* (Niyogi *et al*. 1998) and *szl1npq1* (Li *et al*. 2009). The carotenogenic enzymes impaired in the mutants are shown in Fig. 1. Seeds were stratified in moist soil at 4 °C in the dark for 4 to 5 d before transferring to a climate chamber at 23 °C/18 °C (day/night) air temperature, 60% constant relative air humidity and a 12 h/12 h photoperiod. Photosynthetically active radiation of approx. 60 *µ*mol photons m^−2^ s^−1^ was provided by Osram Fluora Typ L36 W/77 fluorescent tubes (München, Germany) during the day period. Plants were grown in pots (7 × 7 × 8 cm, one plant per pot) containing soil (ED 73 Einheitserde; Balster Einheitserdewerk, Fröndenberg, Germany).

After 3 or 4 weeks from germination, plants of each genotype were divided into two populations (day 0): the first population (‘control’ plants) was kept in the growth light condition of 60 *µ*mol photons m^−2^ s^−1^ while the second population (‘sunfleck’ plants) was transferred to a fluctuating light condition where 20 s pulses of HL (1000 *µ*mol photons m^−2^ s^−1^) were applied with three halogen lamps (Haloline; Osram) every 6 min under the background light intensity of 60 *µ*mol photons m^−2^ s^−1^ during the day. The sunfleck treatment was performed by using an automated set-up described elsewhere (Alter *et al*. 2012).

### Pigment analysis

Following 7 d of the control or sunfleck treatment (day 7), leaf discs (50 mm^2^) were removed from mature leaves that had been fully expanded prior to the starting of the light treatments. All samples were collected in a dark-adapted state at the end of the night period and immediately frozen in liquid nitrogen. They were stored at −80 °C until pigment extraction. For each treatment, five replicates were analysed for wt plants and four for the mutants. Leaf discs were ground in a small amount of liquid nitrogen by using a mortar and a pestle. Pigments were extracted twice in chilled acetone and the final volume of the extract was adjusted to 1 mL. Then the extracts were centrifuged at 15 700 ×*g* for 5 min and syringe filtered prior to the high-performance liquid chromatography (HPLC) analysis.

Photosynthetic pigments were separated by an Allsphere ODS-1 column (5 *µ*m, Alltech Associates, Inc., Deerfield, IL, USA) by using the solvents and protocols modified from Gilmore & Yamamoto (1991). Pigments were identified by retention times and absorption spectra monitored by a Waters 996 photodiode array detector (Waters Corporation, Milford, MA, USA). Peak area was integrated at 440 nm and data were analysed with Waters Empower software.

### Chlorophyll *a* fluorescence analysis

Chlorophyll *a* fluorescence measurements were performed on mature leaves of dark-adapted plants (wt, *n* = 8; mutants, *n* = 4) at the end of the night period of days 0, 1, 3 and 7 by using a portable fluorometer (PAM-2100; Walz, Effeltrich, Germany). Following a measurement of the maximal PSII efficiency, *F*_v_/*F*_m_ = (*F*_m_ – *F*_o_) / *F*_m_, leaves were exposed to actinic light of 600–800 *µ*mol photons m^−2^ s^−1^ for 5 min followed by 2 min of darkness. We noticed that the intensity of the built-in actinic light of PAM-2100 slowly increased from about 600 to 800 *µ*mol photons m^−2^ s^−1^ during the experiment. The maximal intensities of the actinic light reached during the 5 min light induction were: 750–800 *µ*mol photons m^−2^ s^−1^ for wt, *lut2* and *lut5*, and ca. 700 *µ*mol photons m^−2^ s^−1^ for *szl1npq1* and *npq1*. These intensities were saturating for the LL grown plants used in this study. NPQ = (*F*_m_ – *F*_m_’)/*F*_m_’ was determined by applying a train of saturation pulses every 30 s during actinic light illumination and subsequent dark relaxation. The *F*_m_ value of the initial *F*_v_/*F*_m_ measurement was used for NPQ calculation for each plant. Fluorescence nomenclature is according to van Kooten & Snel (1990).

### Sodium dodecyl sulphate–polyacrylamide gel electrophoresis (SDS-PAGE) and immunoblot analysis

For analysis of PsbS protein content, leaf discs (50 mm^2^) were taken from mature leaves of control and sunfleck plants of each genotype on day 7 and immediately frozen in liquid nitrogen. Samples were stored at −80 °C until protein extraction.

Discs were homogenized in an extraction buffer containing 50 mm Tris-HCl (pH 7.6), 7 m urea, 5% SDS, 5% *β*-mercaptoethanol and centrifuged at 15 700 ×*g* for 10 min at 4 °C. An aliquot of the supernatant, containing 2 *µ*g Chl, was loaded on SDS-PAGE. Three replicates were analysed for each genotype and treatment. Together with the samples from the experiments, a reference sample was also loaded on every SDS-PAGE gel. Proteins were blotted onto a nitrocellulose membrane (Roche, Indianapolis, IN, USA) and the PsbS protein was detected with PsbS antiserum (Bonente *et al*. 2008) and alkaline phosphatase-conjugated secondary antibody (Sigma-Aldrich Chemie GmbH, Steinheim, Germany). Band intensity of each sample was quantified by using the ImageJ software (http://rsbweb.nih.gov/ij) and normalized to the intensity of the reference sample of the same membrane.

### Analysis of leaf growth with GROWSCREEN FLUORO

Leaf area growth was analysed by using the GROWSCREEN FLUORO method described by Jansen *et al*. (2009). The experiment for leaf growth analysis was started at about 3 weeks after germination. The projected total leaf area was measured for control and sunfleck plants of each genotype at around 1300 h every other day from day 0 to day 10. At this time of day, leaves of *Arabidopsis* plants are positioned almost horizontally above the soil. The data of the projected total leaf area were fitted to an exponential growth curve:

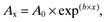
(1)
where *A*_x_ and *A*_0_ are the projected total leaf area on day *x* and day 0, respectively, and *b* the growth factor. Relative growth rate (RGR, % per day) of leaves was obtained by multiplying *b* by 100.

### Analysis of root growth with GROWSCREEN-ROOT

Root system architecture was analysed by using GROWSCREEN-ROOT (Nagel *et al*. 2009). For the analysis of roots, seeds were surface sterilized with sodium hypochlorite solution and sown on sterile agarose (1%, w/w) in Petri dishes (120 × 120 × 17 mm). The medium contained 1/3 modified Hoagland solution [stock solution: 1 m KNO_3_, 1 m Ca(NO_3_)_2_, 1 m MgSO_4_, 1 m KH_2_PO_4_, trace elements]. In the experiment to examine the effect of osmotic shock on root system architecture, 100 mm sorbitol was also added to the medium. Seeds were pushed slightly into the agarose through holes (diameter 2 mm, four holes for four seeds per Petri dish) on the upper side of the closed Petri dishes sealed with fabric tape (Micropore, 3 M Health Care, Neuss, Germany) and placed vertically in a plastic box to avoid light exposure of roots. During germination, the holes were covered with a film (Parafilm, Pechiney Plastic Packaging, Menasha, WI, USA) to keep the seeds moist. This way, the shoot can grow out of the Petri dish (and the plastic box) through the hole, while roots grow in the agarose in the dark (Nagel, Schurr & Walter 2006). The seeds had been stratified in the Petri dishes at 4 °C in the dark for 5 d before they were placed vertically in the plastic box and transferred to the climate chamber with the conditions described previously.

All Petri dishes were kept for 12 d in the control light condition. After this period (day 0), half of the population was exposed to the sunfleck treatment for 5 d, while the other half and the plants growing in Petri dishes containing sorbitol were kept in the control condition. On day 0 and day 5, Petri dishes were placed in an automated carousel device (‘root carousel’ set-up) which is capable of moving up to 70 Petri dishes around. At a distinct corner position where plants are optically accessible, images of the root systems were taken automatically for individual Petri dishes via a high-resolution CCD-camera (IPX-6 M3-TVM, Imperx Inc., Boca Raton, FL, USA) under infrared illumination. Root system architecture of each plant was then analysed by using the GROWSCREEN-ROOT method (for technical details, see Mühlich *et al*. 2008; Nagel *et al*. 2009). Length of the primary root and number of lateral roots were determined for each plant. The RGR of primary root was calculated as:

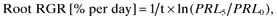
(2)
where t denotes the time interval between two measurement points (i.e. 5 d) and *PRL*_0_ and *PRL*_5_ the primary root length on day 0 and day 5. Likewise, the increase in the number of lateral roots was calculated as:

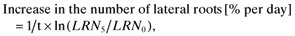
(3)
where *LRN*_0_ and *LRN*_5_ stand for the number of lateral roots on day 0 and day 5.

The number of replicates differed between genotypes and treatments (11–13 for wt; 6–8 for *lut2*; 9–19 for *lut5*; 7–13 for *npq1*; and 7–17 for *szl1npq1*) because of the susceptibility of sorbitol-containing agarose medium (used for the osmotic stress experiment) to fungi contamination in the climate chamber.

### Determination of leaf dry mass

On the last day of the leaf growth analysis (day 10), the above-ground part of plants were harvested for each genotype and treatment (*n* = 12 for wt and *n* = 6 for mutants). Samples were dried in an oven at 70 °C until a constant mass was reached. The dry weight was measured by using an analytical balance (Explorer, Ohaus, Pine Brook, NJ, USA). Leaf mass per area (g per m^2^) was calculated from the dry weight and the projected total leaf area determined for each plant.

### Seed harvesting

Following the experiment of leaf growth analysis, some plants were left under the control and sunfleck conditions. After bolting, inflorescence stems were covered with white paper bags so that only rosette leaves, but not inflorescence and cauline leaves, were directly exposed to the sunflecks. After ca. 20 more weeks to complete flowering and senescence under the corresponding light conditions, plants were moved to LL (20 *µ*mol photons m^−2^ s^−1^) in the same climate chamber and watering was stopped. When they were completely dried, seeds were harvested for each plant separately and weighed with an analytical balance (Explorer, Ohaus). The number of plants used for seed harvesting was: 6 and 5 (control and sunfleck, respectively) for wt, 6 and 3 for *lut2*, 10 and 4 for *lut5*, 7 and 7 for *szl1npq1*, and 7 and 7 for *npq1*. Some plants of *lut2* and *lut5* prematurely died under the sunfleck condition so that fewer plants were available for seed harvesting.

### Statistical data analysis

All data were statistically tested by using SigmaStat (SYSTAT Software GmbH, Erkrath, Germany). A two-way analysis of variance (Tukey test) was used to test differences among genotypes and treatments.

## RESULTS

### Pigment composition

**Figure 2:**
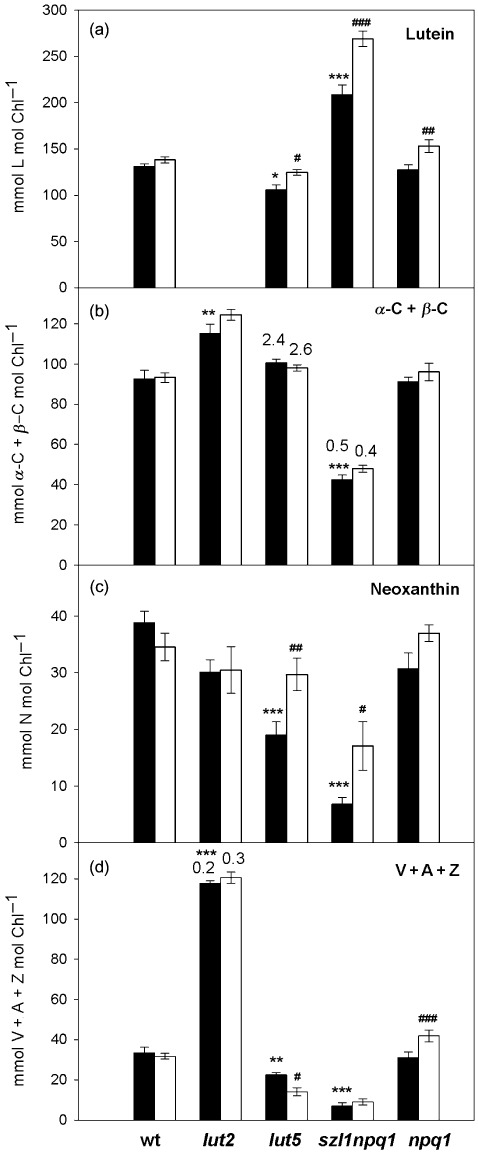
Carotenoid composition in dark-adapted leaves of wild type, *lut2*, *lut5*, *szl1npq1* and *npq1* plants. Samples were harvested at the end of the night period after 7 d of exposure to the growth light environment (control; ca. 60 *µ*mol photons m^−2^ s^−1^; closed bars) or the growth light with high-light pulses (sunfleck; ca. 1000 *µ*mol photons m^−2^ s^−1^ for 20 s every 6 min; open bars). (a) Lutein. (b) Sum of *α*-C and *β*-C. Only *lut5* and *szl1npq1* accumulated more than a trace of *α*-C in leaves. The numbers above the bars of *lut5* and *szl1npq1* indicate the ratio of *α*-C to *β*-C. (c) Neoxanthin. (d) Sum of the V-cycle pigments (V + A + Z). None of the samples contained Z. Antheraxanthin was detected only in *lut2* plants, signifying sustained de-epoxidation of the V-cycle pigments; the numbers above the bars of *lut2* show the de-epoxidation state defined here as (A + Z)/(V + A + Z). Carotenoid contents are given on a Chl basis (mmol mol Chl^−1^). Data are means ± SE (*n* = 5 for wild type and *n* = 4 for the mutants). Significant differences (Tukey test of the two-way anova) between the control and sunfleck treatments are marked with ‘#’ for each plant; significant differences between the control plants of wild type and the mutants are marked with ‘*’ (*P ≤ 0.05, ^#^*P* ≤ 0.05; **P ≤ 0.01, ^##^*P* ≤ 0.01; ***P ≤ 0.001, ^###^*P* ≤ 0.001).

Four-week-old plants of wt, *lut2*, *lut5*, *szl1npq1* and *npq1* were placed under the control or sunfleck condition for 7 d. Figure 2 shows the carotenoid content (relative to Chl) in dark-adapted leaves taken at the end of the night on day 7; these leaves were fully expanded when the treatments were started on day 0. The pigment profiles previously described for these mutants were confirmed in the control plants: lack of L together with increased levels of *β*-C and V + A + Z in *lut2* (Pogson *et al*. 1996); pronounced accumulation of *α*-C at the expense of all xanthophylls and *β*-C (with more than twice as much *α*-C as *β*-C, Fig. 2b) in *lut5* (Fiore *et al*. 2006; Kim & DellaPenna 2006); and over-accumulation of L accompanied by appearance of *α*-C and strong reduction of all *β*-branch carotenoids in *szl1npq1* (Li *et al*. 2009). Under the control condition, the carotenoid composition of *npq1* was very similar to that of wt. Only *lut2* leaves contained A (but not Z) in the control condition even after a 12 h dark period, as indicated by the de-epoxidation state (DPS) of the V-cycle pigments calculated as (A + Z)/(V + A + Z) (Fig. 2d).

The sunfleck treatment did not significantly alter the carotenoid composition in mature leaves of wt and *lut2* plants; only the extent of dark-sustained DPS increased in *lut2* from 0.2 to 0.3 (Fig. 2d), which was attributable to A and not Z. In all other mutants, levels of L significantly increased under the sunfleck condition (Fig. 2a). This increase in L was accompanied by an increase in N, although the change in *npq1* was not statistically significant (Fig. 2c). Generally, carotenes changed little in response to the sunfleck treatment (Fig. 2b). An increase in the V-cycle pigments was found in *npq1* (+35%) while the content of V + A + Z decreased in *lut5* (−37%) under the same condition (Fig. 2d). Following several hours of sunfleck exposure, a trace of Z and somewhat increased amounts of A were detected in leaves of *lut2* (DPS ca. 0.5, data not shown), indicating operation of the V cycle under the sunfleck condition. The V-cycle operation was not seen in other plants under the same condition.

**Figure 3:**
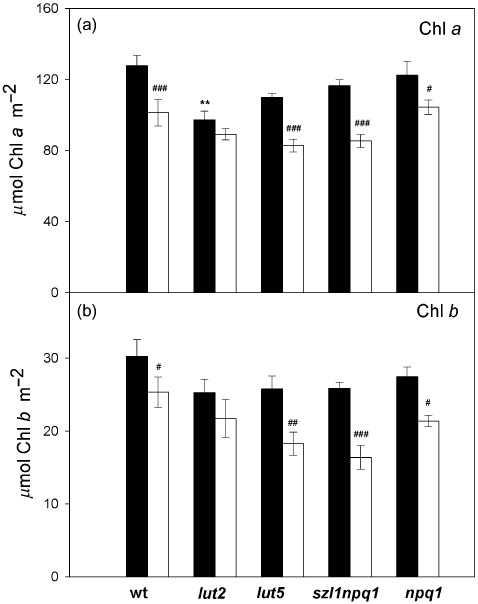
Chlorophyll content of dark-adapted leaves of wild type and the mutants on day 7. Closed bars, control; open bars, sunfleck. For descriptions of the treatments, see legend to Fig. 1. (a) Chlorophyll *a*. (b) Chlorophyll *b*. Chlorophyll concentrations are expressed on a leaf area basis (*µ*mol m^−2^). Data are means ± SE (*n* = 5 for wild type and *n* = 4 for the mutants). Significant differences (Tukey test of the two-way anova) between the two treatments are marked with ‘#’ for each plant; significant differences between the control plants of wild type and the mutants are marked with ‘*’ (^#^*P* ≤ 0.05, ^##^*P* ≤ 0.01, ^###^*P* ≤ 0.001, **P ≤ 0.01).

The Chl *a* and Chl *b* contents per unit leaf area uniformly declined in mature leaves of the five genotypes during the sunfleck treatment (Fig. 3); the decrease was statistically significant for both Chl *a* and Chl *b* in all plants but *lut2*, which had lower Chl contents than wt even under the control condition. Following the 7 d sunfleck treatment, the largest reduction in the total Chl content was found in *lut5* and *szl1npq1*; in these plants Chl *a* declined by nearly 25% and Chl *b* by 30 to 35% compared with the levels found in the corresponding plants in the control condition. The reduction in the Chl content did not significantly alter the ratio of Chl *a* to Chl *b* (Chl *a*/*b*), although the values tended to increase in the mutants under the sunfleck condition. The Chl *a/b* values measured in the control plants on day 7 were 4.3 (±0.2), 3.9 (±0.4), 4.3 (±0.2), 4.5 (±0.2) and 4.5 (±0.3) for wt, *lut2*, *lut5*, *szl1npq1* and *npq1*, respectively. The corresponding values in the sunfleck plants (in the same order) were 4.0 (±0.1), 4.2 (±0.4), 4.6 (±0.2), 5.3 (±0.5) and 4.9 (±0.2).

### The maximal PSII efficiency and NPQ

**Figure 4:**
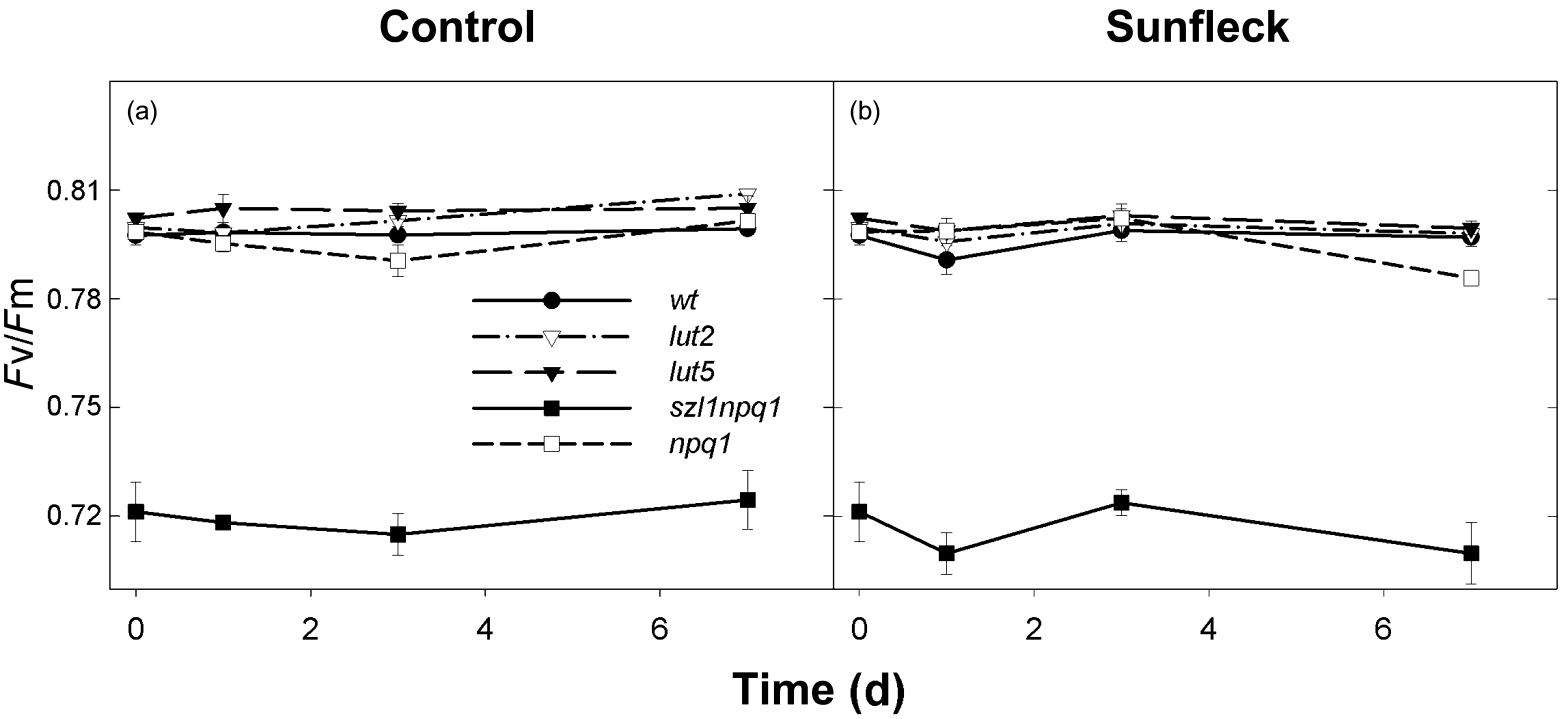
Maximal photosystem II efficiency (*F*_v_/*F*_m_) of dark-adapted leaves of wild type and the mutants during 7 d exposure to the control (a) or sunfleck (b) condition. Measurements were performed at the end of the night period on days 0, 1, 3 and 7. Data are means ± SE (*n* = 8 for wild type and *n* = 4 for the mutants).

The maximal PSII efficiency (*F*_v_/*F*_m_) was measured in mature leaves at the end of the night during the 7 d experiment (Fig. 4). None of the plants exhibited a significant decline in *F*_v_/*F*_m_ in response to the sunfleck treatment; the values remained nearly unchanged under both conditions. The *szl1npq1* always had lower *F*_v_/*F*_m_ of around 0.72, indicating constitutively reduced PSII efficiency.

**Figure 5:**
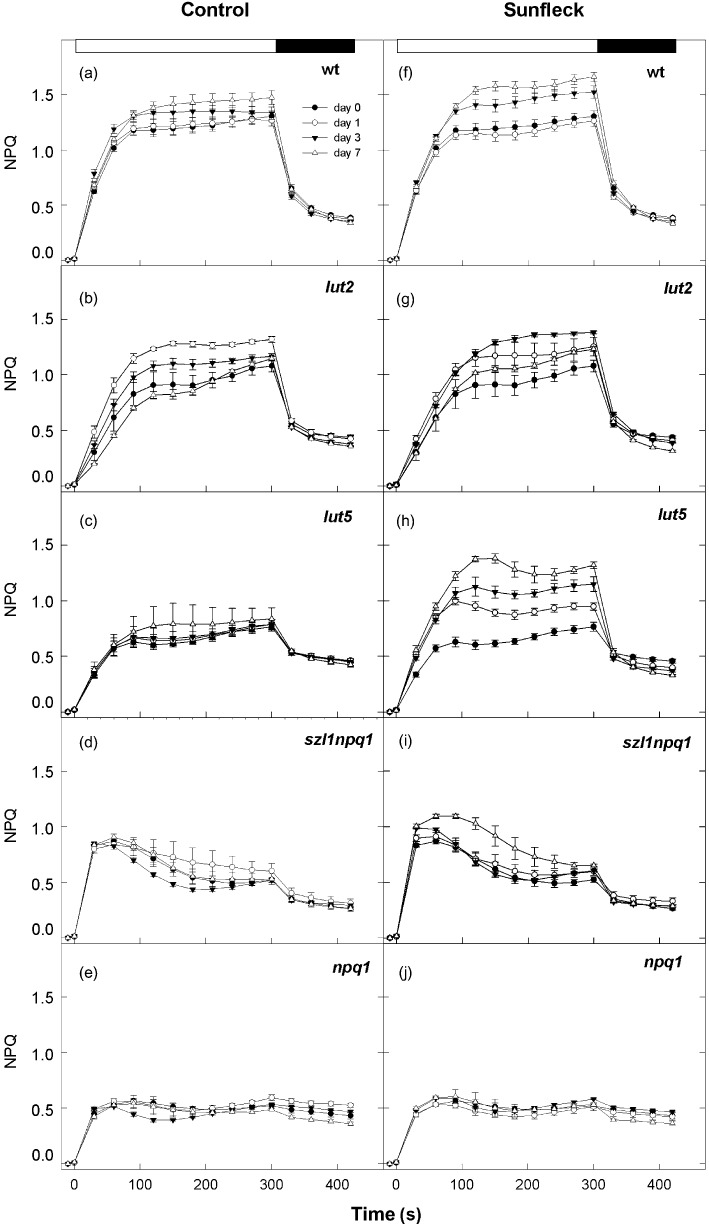
Light induction and dark relaxation of non-photochemical quenching (NPQ) in wild type (a and f), *lut2* (b and g), *lut5* (c and h), *szl1npq1* (d and i) and *npq1* (e and j) plants during 7 d exposure to the control (a–e) or sunfleck (f–j) condition. Light-induction measurements were started immediately after the *F*_v_/*F*_m_ measurements shown in Fig. 4. NPQ was measured during 5 min illumination at saturating light intensities of up to 750–800 *µ*mol photons m^−2^ s^−1^ for wt, *lut2* and *lut5*, and ca. 700 *µ*mol photons m^−2^ s^−1^ for *szl1npq1* and *npq1* (indicated by white bars at the top of the panels a and f) followed by 2 min dark relaxation (indicated by black bars). Data are means ± SE (*n* = 8 for wild type and *n* = 4 for the mutants).

Following the *F*_v_/*F*_m_ measurements, light induction and dark relaxation of NPQ were analysed in the same leaf spots. In the control plants of wt, NPQ rapidly developed to reach 0.6–0.8 in the first 30 s of HL illumination and the maximum of 1.2–1.5 was reached within 5 min (Fig. 5a). Subsequent darkening quickly diminished NPQ to about 0.6 in 30 s, which was followed by a slower decrease to 0.4. The NPQ induction patterns of the control plants of the four mutants (Fig. 5b–e) were comparable with the previous reports. The plants of *lut2* exhibited slower induction of NPQ (Pogson *et al*. 1998) despite retention of a large amount of A (Fig. 2d), reaching no more than 0.2–0.5 in the first 30 s of illumination (Fig. 5b). Likewise, low initial NPQ values were measured in *lut5* which also attained much lower maximal NPQ levels than wt (−50%, Fig. 5c; Dall’Osto *et al*. 2007b). In contrast, the NPQ induction of *szl1npq1* was characterized by a fast rise (Li *et al*. 2009) to ca. 0.8 within 30 s (Fig. 5d); thereafter NPQ did not increase in *szl1npq1*, or even decreased down to 0.4–0.6, during the illumination. The HL exposure induced NPQ of about 0.5 in *npq1* in which the value remained at this level during the subsequent dark period (Fig. 5e; Niyogi *et al*. 1998). Apart from *npq1*, the control plants of *lut5* and *szl1npq1* retained the highest and the lowest NPQ, respectively, at the end of the dark relaxation.

The NPQ capacity increased progressively in the wt plants during the 7 d sunfleck treatment (Fig. 5f). The enhanced NPQ in the sunfleck plants was rapidly reversible upon darkening, indicating that the sunfleck-induced acclimatory NPQ up-regulation was attributable to rapidly inducible and reversible component of NPQ (termed qE). The maximal NPQ levels of *lut2* varied considerably from day to day under both conditions (Fig. 5g) so that effects of the sunfleck treatment could not be evaluated for these plants. The sunfleck plants of *lut5* strongly up-regulated the NPQ capacity already on day 1, followed by a continuous increase until day 7 (Fig. 5h). However, the subsequent darkening always decreased the NPQ to the same low levels within 30 s, suggesting NPQ up-regulation by qE enhancement also in these plants. No or only minor increase in NPQ, respectively, was detected in the sunfleck plants of *npq1* (Fig. 5j) and *szl1npq1* (Fig. 5i) even though the treatment resulted in a large and significant increase in L in both plants (Fig. 2a).

### PsbS protein level

**Figure 6:**
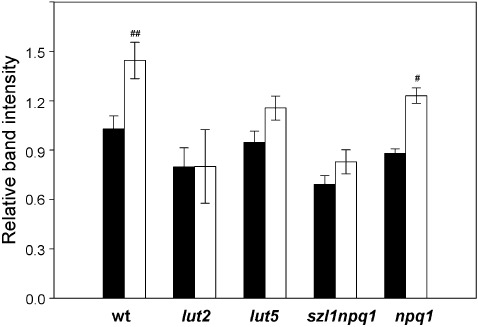
Levels of PsbS protein in leaves of wild type and the mutants. Leaves were collected after 7 d exposure to the control (closed bars) or sunfleck (open bars) condition. Protein samples corresponding to 2 *µ*g total chlorophyll were loaded and separated by SDS-PAGE. PsbS content was determined by Western blot analysis and the band intensity of each sample was normalized to a reference sample. Data are means ± SE (*n* = 3). Significant differences (Tukey test of the two-way anova) between the two treatments are marked with ‘#’ for each plant (^#^*P* ≤ 0.05, ^##^*P* ≤ 0.01). The minor differences between the control plants of wild type and the mutants are not statistically significant.

The levels of PsbS protein, which is essential for qE in higher plants (Li *et al*. 2000), were also analysed in mature leaves of wt and the carotenoid mutants after 7 d in the control or sunfleck condition (Fig. 6). Except in *lut2* which exhibited large variations between individual plants, the PsbS protein content (relative to Chl) increased in all plants under the sunfleck condition; the increase was statistically significant for wt and *npq1* (+40%).

### Growth analyses

**Figure 7:**
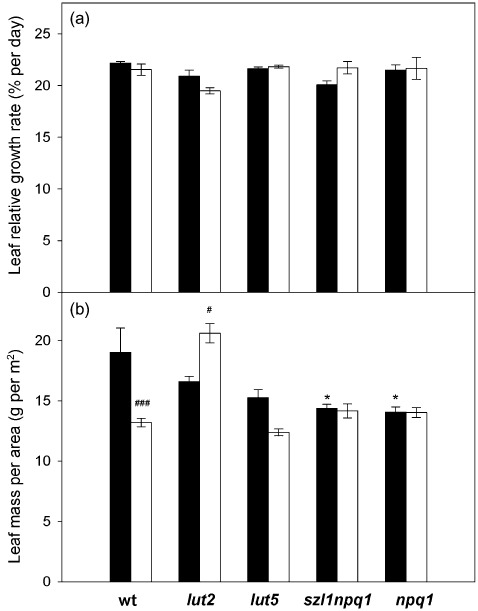
Leaf relative growth rate (RGR) and leaf dry mass per area (LMA) of wild type and the mutants under the control (closed bars) or sunfleck (open bars) condition for 10 d. (a) Leaf RGR. The leaf RGR was obtained by fitting data of the total projected leaf area collected every other day to an exponential growth curve. (b) LMA. LMA was calculated from leaf dry weight and the total projected leaf area measured for each sample on day 10. Values are means ± SE (*n* = 15–37 for leaf RGR analysis and *n* = 6–12 for dry weight measurements). There was no significant difference between the two treatments or genotypes for leaf RGR. For LMA, significant differences (Tukey test of the two-way anova) between the control and sunfleck treatments are marked with ‘#’ for each plant; significant differences between the control plants of wild type and the mutants are marked with ‘*’ (*P ≤ 0.05, ^#^*P* ≤ 0.05, ^###^*P* ≤ 0.001).

**Figure 8:**
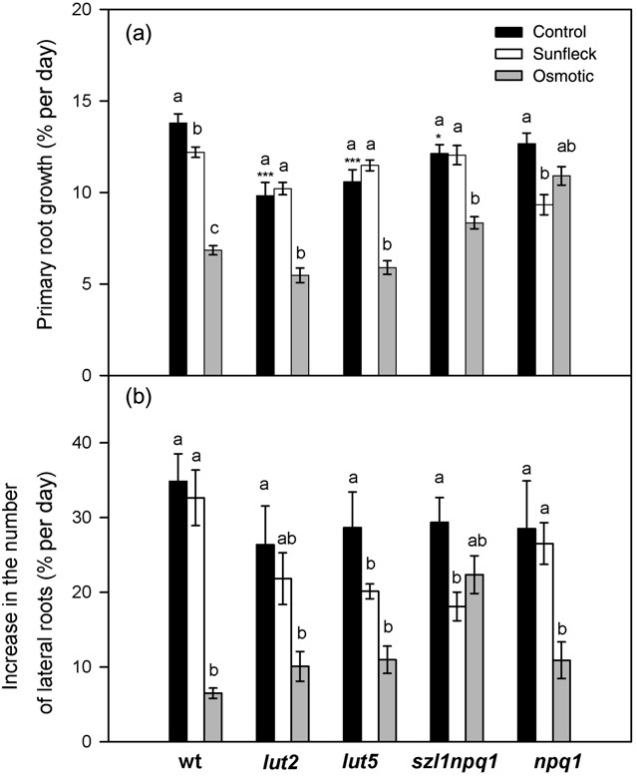
Root growth analysis in wild type and the mutants during the 5 d exposure to the control and sunfleck condition or 17 d cultivation in high-osmorality medium containing 100 mm sorbitol. Relative growth rates of the primary root (a) and lateral roots (b) were calculated from the length of the primary root and the number of lateral roots measured in control (closed bars), sunfleck (open bars) or osmotic stress (grey bars) plants at the beginning (day 0, corresponding to the 12th day of cultivation in the osmotic stress) and at the end of the experiment (day 5, corresponding to the 17th day in the osmotic stress). Values are means ± SE (*n* = 6–19). Significant differences (Tukey test of the two-way anova) between the three treatments are marked with different letters for each plant; significant differences between the control plants of wild type and the mutants are marked with ‘*’ (*P ≤ 0.05, ***P ≤ 0.001).

Leaf and root growth was analysed during 10 d (leaf growth) or 5 d (root growth) of the control or sunfleck treatment (Figs 7 & 8). The RGR calculated from the projected total leaf area was comparable in all plants under both conditions, ranging between 19 and 22% per day (Fig. 7a). A small decrease of leaf RGR observed in wt and *lut2* during the sunfleck treatment, or a minor increase in *szl1npq1*, was statistically not significant; we note that these changes in RGR are most likely underestimated (or overestimated for *szl1npq1*). As the sunfleck treatment induced leaf flattening (i.e. from a convex lamina in the control condition to a flat lamina in the sunfleck condition) in all genotypes, as has been reported by Alter *et al*. (2012), our leaf growth analysis based on projected leaf area results in an underestimation for the control plants, but not for the sunfleck plants. The plants had different leaf dry mass per area (LMA) under the control condition, with the largest LMA found in wt and the lowest in *szl1npq1* and *npq1* (Fig. 7b). While this parameter remained unchanged in the latter two, it decreased in the sunfleck plants of wt and *lut5*. Only *lut2* showed a significant increase in LMA under the sunfleck condition.

For root growth analysis, plants were grown in Petri dishes filled with agarose (Fig. 8). The experiment was stopped after 5 d due to the susceptibility of this cultivation system to fungi attack in non-sterile conditions. Under the control condition, wt and the mutants showed similar rates of lateral root formation (Fig. 8b), whereas the RGR of the primary root was somewhat lower in *lut2*, *lut5* and *szl1npq1* mutants (Fig. 8a). The sunfleck treatment resulted in a significant decrease in the primary root growth of wt and especially *npq1*, while it suppressed formation of lateral roots in *lut5* and *szl1npq1*.

In order to check whether these root growth responses observed under the sunfleck condition are similar to the responses induced by drought or osmotic stress, an additional treatment with 100 mm sorbitol was included in the root growth experiment. The osmotic stress strongly inhibited both primary and lateral root growth in all plants (Fig. 8) although the primary root growth of *npq1* and lateral root formation of *szl1npq1* were affected much less. Overall, the patterns of root growth responses to the osmotic stress were different from the patterns found in the sunfleck plants, suggesting distinct effects and signals of the two treatments.

### Seed production

**Figure 9:**
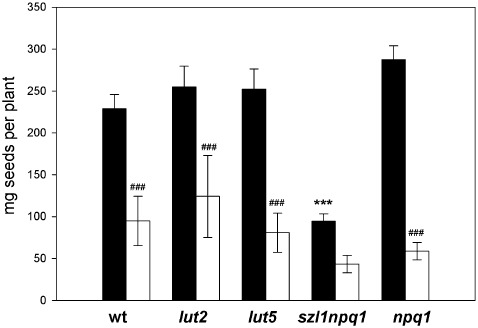
Seed harvest of wild type and mutant plants grown under the control (closed bars) or sunfleck (open bars) condition. Data are means ± SE (*n* = 3–10). Significant differences (Tukey test of the two-way anova) between the control and sunfleck treatments are marked with ‘#’ for each plant; significant differences between wild type and mutants under the control condition are marked with ‘*’ (^###^*P* ≤ 0.001, ***P ≤ 0.001).

Following the leaf growth analysis, the effects of the sunfleck-induced photo-oxidative stress on seed production were examined under the same conditions (Fig. 9). After bolting, inflorescence stems were covered with paper bags so that only rosette leaves and basal cauline leaves were directly exposed to the sunflecks (or the control light). In the control condition, *szl1npq1* produced about 60% less seeds (in weight) than other genotypes. The sunfleck treatment reduced seed yield of all plants, with the largest decrease found in *npq1* (Fig. 9). Note that seed production was analysed after many weeks of exposure to the two conditions. Earlier onset of flowering and rosette leaf senescence observed in all sunfleck plants most likely contributed to the strong reduction of seed yield.

## DISCUSSION

The previous studies have reported the unique pigment phenotypes and photoprotective characteristics of the four mutants (e.g. Pogson *et al*. 1996; Niyogi *et al*. 1998; Dall’Osto *et al*. 2007b; Kalituho *et al*. 2007; Johnson *et al*. 2009; Li *et al*. 2009; Cazzaniga *et al*. 2012). In the present study, we focused on the effects of *α*- and *β*-branch carotenoids and their biosynthesis on photoprotective (NPQ) acclimation and whole-plant growth responses to photo-oxidative stress. Table 1 summarizes the acclimatory responses observed in wt and the carotenoid mutants of *Arabidopsis* during the sunfleck treatment.

**Table 1:** Summary of the acclimatory responses of wild type and the four carotenoid mutants during the sunfleck treatment

	wt	*lut2*	*lut5*	*szl1npq1*	*npq1*
Pigments					
L	No change	–	**Increase**	**Increase**	**Increase**
V + A + Z	No change	No change	**Decrease**	No change	**Increase**
N	No change	No change	**Increase**	**Increase**	No change
*α*-C + *β*-C	No change	No change	No change	No change	No change
Chl	Decrease	Decrease	Decrease	Decrease	Decrease
PSII					
*F*_v_/*F*_m_	No change	No change	No change	No change	No change
NPQ capacity	Increase	**Variable**	Increase	**Little change**	**No change**
PsbS	Increase	**Variable**	Increase	Increase	Increase
Growth					
Leaf RGR	No change	No change	No change	No change	No change
LMA	Decrease	**Increase**	Decrease	**No change**	**No change**
Root RGR	Decrease in primary root	**No change**	**Decrease in lateral roots**	**Decrease in lateral roots**	Decrease in primary root
Seed harvest	Decrease	Decrease	Decrease	Decrease	Decrease

Responses in the mutant genotypes that were different from wt are highlighted.

L, lutein; V, violaxanthin; A, antheraxanthin; Z, zeaxanthin; N, neoxanthin; *α*-C, *α*-carotene; *β*-C, *β*-carotene; PSII, photosystem II; NPQ, non-photochemical quenching; RGR, relative growth rate; LMA, leaf dry mass per area.

### Acclimatory adjustment of pigment composition

A marked response of *Arabidopsis* to strong sunflecks is the decrease in leaf Chl content (Fig. 3; Yin & Johnson 2000; Alter *et al*. 2012). Unlike in the study by Alter *et al*. (2012), in which more severe sunflecks resulted in an increase in the V-cycle pigments in wt, Chl-based carotenoid contents did not change in mature leaves of wt in the present work (Fig. 2), indicating net degradation of Chl and all carotenoids under the photo-oxidative condition. In contrast, exposure to HL and low temperature did not enhance xanthophyll degradation in *Arabidopsis* leaves (Ramel *et al*. 2012a); only the *β*-C oxidation increased, which was interpreted as chemical scavenging of PSII-derived ^1^O_2_ by *β*-C. Whether the sunfleck-induced acclimatory decline in Chl and carotenoid contents is a result of chemical reactions with ROS or enzymatic degradation is a question for future investigations.

Unlike in wt, the decrease in Chl was accompanied by an increase in L and N in *lut5* and *szl1npq1* or L and V in *npq1* (Fig. 2). The *lut5* and *szl1* alleles are characterized by enhanced synthesis of *α*-branch carotenoids (Fig. 2; Fiore *et al*. 2006; Kim & DellaPenna 2006; Li *et al*. 2009) and accumulation of *α*-C. This kind of pigment phenotype is found in leaves of tropical plants under shade environments (e.g. Thayer & Björkman 1990; Logan *et al*. 1997; Matsubara *et al*. 2009). The sunfleck plants of *lut5* and *szl1npq1* showed the largest decrease in Chl contents (Fig. 3), suggesting that high *α*/*β*-ratios may exacerbate photo-oxidative stress (Dall’Osto *et al*. 2007b). Higher turnover of *α*-C than *β*-C in light (Beisel *et al*. 2010) could also increase the sensitivity of these plants to HL (Kim *et al*. 2009). Increased L and N accumulation observed in the sunfleck plants of *lut5* and *szl1npq1* (Fig. 2) may be an acclimatory response to compensate in part for the low availability of V + A + Z. Both L and N are able to contribute to photoprotection via quenching of singlet or triplet Chl (L) (Dall’Osto *et al*. 2006; Matsubara *et al*. 2008, 2011; Li *et al*. 2009) and detoxification of ROS (L and N) (Peng *et al*. 2006; Dall’Osto *et al*. 2007a).

The control plants of *npq1* had a near-wt carotenoid composition (Fig. 2). The major difference between wt and *npq1* resides in the inability of the latter to form Z through light-induced de-epoxidation of V (Niyogi *et al*. 1998). Thus, wt and *npq1* would display similar phenotypes unless V de-epoxidation occurs in wt. The distinct pigment changes found in these plants (Fig. 2) suggest that the sunfleck treatment induced V de-epoxidation in wt, although neither A nor Z was detected in leaves after several hours of sunfleck exposure (data not shown). A small amount of A + Z formed during the 20 s sunflecks may have been converted back to V in the sunfleck intervals. The lack of the V-cycle operation and Z-associated photoprotection in *npq1* probably intensified the photo-oxidative stress (Havaux & Niyogi 1999) and promoted synthesis of V and L (Fig. 2). A HL-induced parallel increase in V + A + Z and L has been seen in leaves of different species, including *Arabidopsis* (Beisel *et al*. 2010) and *Persea americana* Mill. (Förster, Osmond & Pogson 2009).

The extent of the sunfleck-induced increase in *α*- and *β*-branch xanthophylls in the different mutants corresponds by and large to the genotype-specific balance between *α*- and *β*-branch carotenoids (Fig. 2), which is determined by the activities of cyclases and hydroxylases. This suggests the importance of these enzymes in controlling stress-induced acclimatory adjustment of leaf xanthophyll composition. Indeed, HL induces up-regulation of a gene encoding non-haeme di-iron *β*-ring hydroxylase in *Arabidopsis* plants (Cuttriss *et al*. 2007), which may represent an early step to trigger a stress-induced increase in V + A + Z.

While most of the xanthophylls are bound in the antenna complexes, carotenes are in the core complex of PSII and PSI (Yamamoto & Bassi 1996). The *lut2* mutant had somewhat more *β*-C per Chl than wt, whereas *szl1npq1* had very low carotene contents (Fig. 2b). Interestingly, some of the carotene binding sites of the PSII and PSI core complexes are empty in the *szl1* mutant (Cazzaniga *et al*. 2012), which may be related to the low *F*_v_/*F*_m_ of *szl1npq1* (Fig. 4). Because the sunflecks did not significantly affect the carotene profile of wt and the mutants (Fig. 2b), Chl and carotenes must have decreased to similar extents during acclimation, regardless of the carotenoid compositions (Fig. 2) and changes in NPQ (Fig. 5). When *α*-C naturally occurs in a large amount in leaves, the balance between *α*-C and *β*-C shifts during acclimation to light environments (e.g. Thayer & Björkman 1990; Logan *et al*. 1997; Matsubara *et al*. 2009). No such shift was found in *lut5* and *szl1npq1* (Fig. 2b), demonstrating their inability to adjust carotene balance.

Together, *α*- and *β*-branch carotenoid composition seems to determine the sensitivity of *Arabidopsis* leaves to photo-oxidative stress, which in turn determines the extent of acclimatory pigment degradation and feedback up-regulation of xanthophyll biosynthesis.

### Acclimatory up-regulation of NPQ

Dynamic photoprotection by thermal energy dissipation, which is regulated by formation of a pH gradient across the thylakoid membrane (ΔpH), plays a crucial role in plants exposed to sunflecks (Logan *et al*. 1997; Watling *et al*. 1997; Adams *et al*. 1999). During the 7 d sunfleck treatment, the NPQ capacity increased in wt (Fig. 5f), concomitant with a significant increase in the PsbS protein content relative to Chl (Fig. 6); the PsbS protein is needed for the ΔpH-dependent, qE component of NPQ in higher plants (Li *et al*. 2000), and its absence reduces fitness of *Arabidopsis* plants under fluctuating light (Külheim, Ågren & Jansson 2002; Logan, Terry & Niyogi 2008). An increase in PsbS was also found in the carotenoid mutants following the sunfleck treatment, with an exception of *lut2* showing large variations in PsbS level (Fig. 6). The fact that a 40% increase in PsbS did not allow NPQ enhancement in *npq1* (Fig. 5j) underscores the involvement of both PsbS and Z in NPQ.

The control plants of *lut2*, *lut5* and *npq1* had lower NPQ than wt (Fig. 5a–c,e), in agreement with the NPQ characteristics previously described in these mutants (Niyogi *et al*. 1998; Pogson *et al*. 1998; Dall’Osto *et al*. 2007b). Conversely, *szl1npq1* achieved somewhat higher NPQ in the initial induction phase (Fig. 5d), which has been attributed to an extra pool of L (Li *et al*. 2009). Rapid NPQ induction with additional L has also been documented in *Arabidopsis* plants overexpressing *ε*-cyclase (Pogson & Rissler 2000) or in species having the Lx cycle (Matsubara *et al*. 2008, 2011,; Esteban *et al*. 2010; Förster *et al*. 2011). The lack of L in *lut2* (Fig. 2a), on the other hand, slows down the NPQ induction (Pogson *et al*. 1998) even in the presence of A (Fig. 2d), and seems to cause erratic behaviour of NPQ (Fig. 5b,g) presumably through destabilization of trimeric LHCII (Lokstein *et al*. 2002) and variable PsbS level (Fig. 6).

The most pronounced acclimatory qE enhancement was found in the sunfleck plants of *lut5* (Fig. 5h) which had a reduced NPQ capacity under the control condition (Fig. 5c; Dall’Osto *et al*. 2007b). The NPQ in *lut5* may be limited by its small V + A + Z content (Fig. 2d) as the PsbS levels were comparable in *lut5* and wt (Fig. 6; Dall’Osto *et al*. 2007b). The marked qE up-regulation, despite diminishing V + A + Z found in the sunfleck plants of *lut5*, may be explained by the increased accumulation of L, N and/or PsbS (Figs 2 & 6), although a significant increase in L and N as well as PsbS had little or no influence on acclimatory up-regulation of NPQ in *szl1npq1* or *npq1* (Fig. 5i,j). The lack of NPQ increase despite the substantial accumulation of additional L (+25 mmol mol Chl^−1^) is at variance with rescuing of the qE-deficient *npq1* phenotype in *szl1npq1* (Li *et al*. 2009). Unlike Z, the quenching effect of L is not unequivocal; for example, substitution of V by L (or Lx by L) in one of the xanthophyll binding sites results in fluorescence quenching in monomeric recombinant antenna complexes of PSII (Formaggio, Cinque & Bassi 2001; Matsubara *et al*. 2007; Li *et al*. 2009), but not in recombinant Lhcb1 or native LHCII trimers in the case of Lx-L substitution (Matsubara *et al*. 2007). Multiple pools of L, having different localization and hence different functions, are probably responsible for these seemingly contradictory observations concerning the quenching effect of L.

Notably, the plants of *szl1npq1* were unable to maintain high NPQ following rapid induction; NPQ started to decline after 1 min in HL and only half of the initial level remained at the end of the 5 min illumination (Fig. 5d,i). This peculiar pattern of NPQ induction, observed not only in dark-adapted plants but also in light-adapted plants under different light intensities except for very strong light (Matsubara & Osmond, unpublished data), contrasts with the typical NPQ induction curve previously reported in this mutant (Li *et al*. 2009). In our experimental conditions, the unique pigment phenotype of *szl1npq1*, that is, extremely rich in L and poor in all other xanthophylls and carotenes (Fig. 2), and/or a 50% decrease in the two minor antenna complexes CP29 and CP26 (Li *et al*. 2009), may have interfered with qE stabilization while allowing rapid ΔpH and qE formation (Fig. 5d,i). The qE component of NPQ involves PsbS- and Z-dependent protein conformational changes which supposedly result in dissociation of PSII antenna supercomplexes consisting of CP29, CP24 and LHCII (Betterle *et al*. 2009). The *szl1npq1* mutant offers an interesting system to study the mechanism of qE stabilization and roles of Z and L therein.

Based on these observations, we conclude that the sunfleck-induced acclimatory up-regulation of NPQ by qE enhancement necessitates the ability to synthesize Z by de-epoxidation of V; neither in *szl1npq1* nor in *npq1* can additional L molecules replace Z in this acclimatory function. Thus, the composition of de-epoxidized *α*- and *β*-branch xanthophylls affects not only the intensity and kinetics of NPQ but also the ability and capacity of plants to adjust the maximal NPQ level during acclimation to stress conditions.

### Acclimation via growth

In a wide range of plant species, LMA is strongly correlated with the daily total irradiance received by leaves (Poorter *et al*. 2009); sun-exposed leaves usually have higher LMA than leaves growing in the shade (e.g. Matsubara *et al*. 2009). The decrease in LMA found in the sunfleck plants of wt and possibly also *lut5* (Fig. 7b), despite the greater daily total irradiance under the sunfleck condition than the control condition, may be due to the enhanced NPQ in these plants (Fig. 5f,h), resulting in lower light energy utilization for photosynthesis. Only *lut2* plants, having no *α*-branch carotenoids and extremely large amounts of V + A + Z which were always partially de-epoxidized (Fig. 2), exhibited an increase in LMA (Fig. 7b), indicating that their leaves became thicker and/or had higher dry mass density under the sunfleck condition. Although reduced leaf area growth has been reported previously for *lut2* compared with wt and *npq1* (Kalituho *et al*. 2007), we found comparable leaf RGR values in these three plants (as well as *lut5* and *szl1npq1*) under both control and sunfleck conditions (Fig. 7a).

Compared with chloroplasts and leaves, much less is known about physiological functions and stress responses of the carotenoid biosynthetic pathway for non-photosynthetic organs (Howitt & Pogson 2006; Cazzonelli 2011). The plants of *szl1npq1* produced far less seeds than the others in the control condition (Fig. 9) even though leaf and root growth did not show such inhibition (Figs 7 & 8). This may indicate a direct effect of the altered carotenoid biosynthesis on the reproductive organ of *szl1npq1*. The long-term sunfleck treatment further diminished the seed yield of *szl1npq1*, as seen also in other genotypes, demonstrating a strong negative impact of photo-oxidative stress. As for roots, our root growth analysis revealed different sensitivity to the sunflecks among the five genotypes, as well as between the primary and lateral roots within a genotype (Fig. 8). The primary root growth decreased in the sunfleck plants having normal *α*/*β*-ratios in leaves (wt and *npq1*), whereas those having high *α*/*β*-ratios (*lut5* and *szl1npq1*) responded by reducing lateral root formation (Figs 2 & 8). Were these distinct responses of root growth caused by different sensitivity (of leaves) to photo-oxidative stress and/or by altered carotenoid metabolism?

Two plant hormones are known to be derived from *β*-branch carotenoids via the activities of carotenoid cleavage dioxygenases: ABA from 9′-*cis* N or 9′-*cis* V (Tan *et al*. 1997; Nambara & Marion-Poll 2005), and SL from *β*-C (Schwartz, Qin & Loewen 2004; Gomez-Roldan *et al*. 2008; Umehara *et al*. 2008). In addition to controlling aboveground growth, for example, bud dormancy/outgrowth, leaf expansion and shoot branching (Koornneef *et al*. 2002; Horvath *et al*. 2003; Kozuka *et al*. 2005; Gomez-Roldan *et al*. 2008; Umehara *et al*. 2008), both ABA and SL can suppress lateral root formation under drought and osmotic stress (ABA) or phosphate deficiency (SL) (De Smet *et al*. 2006; Koltai 2011). Alterations in carotenoid biosynthesis can affect apocarotenoid levels, as exemplified by the *aba1* (*npq2*) mutant lacking Z epoxidase and thus also epoxycarotenoids and ABA (Koornneef *et al*. 1982), or the *β*-hydroxylase-deficient mutants having less ABA in leaves (Tian, DellaPenna & Zeevaart 2004).

Conceivably, the *α*- and *β*-branch activities may affect plant growth behaviour via apocarotenoid hormones. For the acclimatory responses of LMA and root growth, however, there seems to be an interaction between carotenoid metabolism and photo-oxidative stress because the phenotypic differences in LMA and root growth emerged only in the sunfleck plants, not in the control plants (Figs 7b & 8). As short sunflecks primarily cause photo-oxidative stress in *Arabidopsis* leaves (Alter *et al*. 2012), stress signals may travel from leaves to roots. The signals of sunflecks are different from those of osmotic stress, judging by the distinct patterns of root growth under these conditions (Fig. 8). As growing roots quickly respond to carbon supply from the shoot (Nagel *et al*. 2006; Yazdanbakhsh & Fisahn 2010), the sunfleck treatment may have affected the root growth by decreasing leaf carbohydrate status. Furthermore, oxidative signals involving ROS (Karpinski *et al*. 1999; Rossel *et al*. 2007) are implicated in long-distance signalling in systemic HL acclimation. As *α*- and *β*-branch xanthophylls have different ROS-scavenging capacities in leaves (Dall’Osto *et al*. 2007b; Havaux *et al*. 2007; Johnson *et al*. 2007), altered balance between the two branches could influence long-distance stress signalling indirectly via its effect on ROS detoxification. Moreover, a recent study by Ramel *et al*. (2012b), which has shown reprogramming of gene transcription by volatile oxidation products of *β*-C (especially *β*-cyclocitral), suggests a direct role of *β*-C as a precursor of messenger molecules in ^1^O_2_-induced systemic acclimation. The distinct changes in LMA and root growth found in the carotenoid mutants during the sunfleck treatment (Figs 7 & 8) provide a further support to carotenoid functions in whole-plant acclimation to photo-oxidative stress.

## CONCLUSIONS

Alterations in the balance between the *α*- and *β*-branch of the carotenoid biosynthetic pathway can affect not only acclimatory adjustment of pigment composition and photoprotection in leaves but also photo-oxidative stress responses of leaf and root growth in *Arabidopsis* plants. Except for the very low seed yield of *szl1npq1*, however, large variations in carotenoid biosynthesis did not seriously penalize any of the mutants under our experimental conditions, demonstrating the ability of *Arabidopsis* to cope, to certain extent, with perturbations in the pathway through acclimation. Nevertheless, the conserved leaf pigment complement suggests strong selection pressure favouring the normal carotenoid composition and acclimatory regulation at least in chloroplasts. Further investigations are needed to elucidate the roles of *α*- and *β*-branch carotenoid metabolism in whole-plant acclimation to variable and adverse environments.

## References

[b1] Adams WW, Demmig-Adams B, Logan BA, Barker DH, Osmond CB (1999). Rapid changes in xanthophyll cycle-dependent energy dissipation and photosystem II efficiency in two vines, *Stephania japonica* and *Smilax australis*, growing in the understory of an open *Eucalyptus* forest. Plant, Cell & Environment.

[b2] Alter P, Dreissen A, Luo F-L, Matsubara S (2012). 10.1007/s11120-012-9757-2.

[b3] Beisel KG, Jahnke S, Hofmann D, Köppchen S, Schurr U, Matsubara S (2010). Continuous turnover of carotenes and chlorophyll a in mature leaves of *Arabidopsis* revealed by ^14^CO_2_ pulse-chase labeling. Plant Physiology.

[b4] Betterle M, Ballottari M, Zorzan S, de Bianchi S, Cazzaniga S, Dall’Osto L, Morosinotto T, Bassi R (2009). Light-induced dissociation of an antenna hetero-oligomer is needed for non-photochemical quenching induction. Journal of Biological Chemistry.

[b5] Bonente G, Passarini F, Cazzaniga S, Mancone C, Buia MC, Tripodi M, Bassi R, Caffarri S (2008). The occurrence of the *psbS* gene product in *Chlamydomonas reinhardtii* and in other photosynthetic organisms and its correlation with energy quenching. Photochemistry and Photobiology.

[b6] Cazzaniga S, Li Z, Niyogi KK, Bassi R, Dall’Osto L (2012). The *Arabidopsis szl1* mutant reveals a critical role of *β*-carotene in photosystem I photoprotection. Plant Physiology.

[b7] Cazzonelli CI (2011). Carotenoids in nature: insights from plants and beyond. Functional Plant Biology.

[b8] Cuttriss AJ, Chubb AC, Alawady A, Grimm B, Pogson BJ (2007). Regulation of lutein biosynthesis and prolamellar body formation in *Arabidopsis*. Functional Plant Biology.

[b11] Dall’Osto L, Lico C, Alric J, Giuliano G, Havaux M, Bassi R (2006). Lutein is needed for efficient chlorophyll triplet quenching in the major LHCII antenna complex of higher plants and effective photoprotection *in vivo* under strong light. BMC Plant Biology.

[b9] Dall’Osto L, Cazzaniga S, North H, Marion-Poll A, Bassi R (2007a). The *Arabidopsis aba4-1* mutant reveals a specific function for neoxanthin in protection against photooxidative stress. The Plant Cell.

[b10] Dall’Osto L, Fiore A, Cazzaniga S, Giuliano G, Bassi R (2007b). Different roles of *α*- and *β*-branch xanthophylls in photosystem assembly and photoprotection. Journal of Biological Chemistry.

[b12] De Smet I, Zhang H, Inzé D, Beeckman T (2006). A novel role for abscisic acid emerges from underground. Trends in Plant Science.

[b13] DellaPenna D, Pogson BJ (2006). Vitamin synthesis in plants: tocopherols and carotenoids. Annual Review of Plant Biology.

[b14] Esteban R, Matsubara S, Jiménez MS, Morales D, Brito P, Lozenzo R, Fernández-Marin B, Becerril JM, García-Plazaola JI (2010). Operation and regulation of the lutein epoxide cycle in seedlings of *Ocotea foetens*. Functional Plant Biology.

[b15] Evans JR, Poorter H (2001). Photosynthetic acclimation of plants to growth irradiance: the relative importance of specific leaf area and nitrogen partitioning in maximizing carbon gain. Plant, Cell & Environment.

[b16] Fiore A, Dall’Osto L, Fraser PD, Bassi R, Giuliano G (2006). Elucidation of the *β*-carotene hydroxylation pathway in *Arabidopsis thaliana*. FEBS Letters.

[b17] Formaggio E, Cinque G, Bassi R (2001). Functional architecture of the major light-harvesting complex from higher plants. Journal of Molecular Biology.

[b18] Förster B, Osmond CB, Pogson BJ (2009). De novo synthesis and degradation of Lx and V cycle pigments during shade and sun acclimation in avocado leaves. Plant Physiology.

[b19] Förster B, Pogson BJ, Osmond CB (2011). Lutein from de-epoxidation of lutein epoxide replaces zeaxanthin to sustain an enhanced capacity for non-photochemical chlorophyll fluorescence quenching in avocado shade leaves in the dark. Plant Physiology.

[b20] Franklin KA (2008). Shade avoidance. New Phytologist.

[b21] García-Plazaola JI, Matsubara S, Osmond CB (2007). The lutein epoxide cycle in higher plants: its relationships to other xanthophyll cycles and possible functions. Functional Plant Biology.

[b22] Gilmore AM, Yamamoto HY (1991). Resolution of lutein and zeaxanthin using a non-endcapped, lightly carbon-loaded C_18_ high-performance liquid chromatographic column. Journal of Chromatography.

[b23] Gomez-Roldan V, Fermas S, Brewer PB (2008). Strigolactone inhibition of shoot branching. Nature.

[b25] Havaux M, Niyogi KK (1999). The violaxanthin cycle protects plants from photooxidative damage by more than one mechanism. Proceedings of the National Academy of Sciences of the United States of America.

[b24] Havaux M, Dall’Osto L, Bassi R (2007). Zeaxanthin has enhanced antioxidant capacity with respect to all other xanthophylls in *Arabidopsis* leaves and functions independent of binding to PSII antennae. Plant Physiology.

[b26] Horvath DP, Anderson JV, Chao WS, Foley ME (2003). Knowing when to grow: signals regulating bud dormancy. Trends in Plant Science.

[b27] Howitt CA, Pogson BJ (2006). Carotenoid accumulation and function in seeds and non-green tissues. Plant, Cell & Environment.

[b28] Jansen M, Gilmer F, Biskup B (2009). Simultaneous phenotyping of leaf growth and chlorophyll fluorescence via GROWSCREEN FLUORO allows detection of stress tolerance in *Arabidopsis thaliana* and other rosette plants. Functional Plant Biology.

[b29] Johnson MP, Havaux M, Triantaphylidès C, Ksas B, Pascal AA, Robert B, Davison PA, Ruban AV, Horton P (2007). Elevated zeaxanthin bound to oligomeric LHCII enhances the resistance of *Arabidopsis* to photooxidative stress by a lipid-protective, antioxidant mechanism. Journal of Biological Chemistry.

[b30] Johnson MP, Pérez-Bueno ML, Zia A, Horton P, Ruban AV (2009). The zeaxanthin-independent and zeaxanthin-dependent qE components of nonphotochemical quenching involve common conformational changes within the photosystem II antenna in *Arabidopsis*. Plant Physiology.

[b31] Kalituho L, Rech J, Jahns P (2007). The roles of specific xanthophylls in light utilization. Planta.

[b32] Karpinski S, Reynolds H, Karpinska B, Wingsle G, Creissen G, Mullineaux P (1999). Systemic signaling and acclimation in response to excess excitation energy in *Arabidopsis*. Science.

[b33] Kim J, DellaPenna D (2006). Defining the primary route for lutein synthesis in plants: the role of *Arabidopsis* carotenoid *β*-ring hydroxylase CYP97A3. Proceedings of the National Academy of Sciences of the United States of America.

[b34] Kim J, Smith JJ, Tian L, DellaPenna D (2009). The evolution and function of carotenoid hydroxylases in *Arabidopsis*. Plant and Cell Physiology.

[b35] Koltai H (2011). Strigolactones are regulators of root development. New Phytologist.

[b37] Koornneef M, Jorna ML, Brinkhorst-van der Swan DLC, Karssen CM (1982). The isolation of abscisic acid (ABA) deficient mutants by selection of induced revertants in non-germinating gibberellins sensitive lines of *Arabidopsis thaliana* (L.) Heynh. Theoretical and Applied Genetics.

[b36] Koornneef M, Bentsink L, Hilhorst H (2002). Seed dormancy and germination. Current Opinion in Plant Biology.

[b72] van Kooten O, Snel JFH (1990). The use of chlorophyll fluorescence nomenclature in plant stress physiology. Photosynthesis Research.

[b38] Kozuka T, Horiguchi G, Kim G-T, Ohgishi M, Sakai T, Tsukaya H (2005). The different growth responses of the *Arabidopsis thaliana* leaf blade and the petiole during shade avoidance are regulated by photoreceptors and sugar. Plant and Cell Physiology.

[b39] Krause GH, Koroleva OY, Dalling JW, Winter K (2001). Acclimation of tropical tree seedlings to excessive light in simulated tree-fall gaps. Plant, Cell & Environment.

[b40] Külheim C, Ågren J, Jansson S (2002). Rapid regulation of light harvesting and plant fitness in the field. Science.

[b42] Li X-P, Björkman O, Shih C, Grossman AR, Rosenquist M, Jansson S, Niyogi KK (2000). A pigment-binding protein essential for regulation of photosynthetic light harvesting. Nature.

[b41] Li Z, Ahn TK, Avenson TJ, Ballottari M, Cruz JA, Kramer DM, Bassi R, Fleming GR, Keasling JD, Niyogi KK (2009). Lutein accumulation in the absence of zeaxanthin restores nonphotochemical quenching in the *Arabidopsis thaliana npq1* mutant. The Plant Cell.

[b43] Logan BA, Barker DH, Adams WW, Demmig-Adams B (1997). The response of xanthophyll cycle-dependent energy dissipation in *Alocasia brisbanensis* to sunflecks in a subtropical rainforest. Australian Journal of Plant Physiology.

[b44] Logan BA, Terry SG, Niyogi KK (2008). *Arabidopsis* genotypes with differing levels of *psbS* expression differ in photosystem II quantum yield, xanthophyll cycle pool size, and aboveground growth. International Journal of Plant Sciences.

[b45] Lokstein H, Tian L, Polle JEW, DellaPenna D (2002). Xanthophyll biosynthetic mutants of *Arabidopsis thaliana*: altered nonphotochemical quenching of chlorophyll fluorescence is due to changes in photosystem II antenna size and stability. Biochimica et Biophysica Acta.

[b49] Matsubara S, Morosinotto T, Osmond CB, Bassi R (2007). Short- and long-term operation of the lutein-epoxide cycle in light-harvesting antenna complexes. Plant Physiology.

[b48] Matsubara S, Krause GH, Seltmann M, Virgo A, Kursar TA, Jahns P, Winter K (2008). Lutein epoxide cycle, light harvesting and photoprotection in species of the tropical tree genus *Inga*. Plant, Cell & Environment.

[b47] Matsubara S, Krause GH, Aranda J, Virgo A, Beisel KG, Jahns P, Winter K (2009). Sun-shade patterns of leaf carotenoid composition in 86 species of neotropical forest plants. Functional Plant Biology.

[b46] Matsubara S, Chen Y-C, Caliandro R, Govindjee, Clegg RM (2011). Photosystem II fluorescence lifetime imaging in avocado leaves: contributions of the lutein-epoxide and violaxanthin cycles to fluorescence quenching. Journal of Photochemistry and Photobiology B: Biology.

[b50] Mühlich M, Truhn D, Nagel KA, Walter A, Scharr H, Aach T, Rigoll G (2008). Measuring plant root growth. Lecture Notes in Computer Science 5096.

[b51] Müller P, Li X-P, Niyogi KK (2001). Non-photochemical quenching. A response to excess light energy. Plant Physiology.

[b53] Nagel KA, Schurr U, Walter A (2006). Dynamics of root growth stimulation in *Nicotiana tabacum* in increasing light intensity. Plant, Cell & Environment.

[b52] Nagel KA, Kastenholz B, Jahnke S (2009). Temperature response of roots: impact on growth, root system architecture and implications for phenotyping. Functional Plant Biology.

[b54] Nambara E, Marion-Poll A (2005). Abscisic acid biosynthesis and catabolism. Annual Review of Plant Biology.

[b55] Niyogi KK, Grossman AR, Björkman O (1998). *Arabidopsis* mutants define a central role for the xanthophyll cycle in the regulation of photosynthetic energy conversion. The Plant Cell.

[b57] Pearcy RW (1990). Sunfleck and photosynthesis in plant canopies. Annual Review of Plant Physiology and Plant Molecular Biology.

[b56] Peng C, Lin Z, Su Y, Lin G, Dou H, Zhao C (2006). The antioxidative function of lutein: electron spin resonance studies and chemical detection. Functional Plant Biology.

[b60] Pogson BJ, Rissler HM (2000). Genetic manipulation of carotenoid biosynthesis and photoprotection. Philosophical Transactions of the Royal Society B: Biological Sciences.

[b58] Pogson BJ, McDonald KA, Truong M, Britton G, DellaPenna D (1996). *Arabidopsis* carotenoid mutants demonstrate that lutein is not essential for photosynthesis in higher plants. The Plant Cell.

[b59] Pogson BJ, Niyogi KK, Björkman O, DellaPenna D (1998). Altered xanthophyll compositions adversely affect chlorophyll accumulation and nonphotochemical quenching in *Arabidopsis* mutants. Proceedings of the National Academy of Sciences of the United States of America.

[b61] Poorter H, Niinemets Ü, Poorter L, Wright IJ, Villar R (2009). Causes and consequences of variation in leaf mass per area (LMA): a meta-analysis. New Phytologist.

[b62] Poorter H, Niklas KJ, Reich PB, Oleksyn J, Poot P, Mommer L (2012). Biomass allocation to leaves, stems and roots: meta-analyses of interspecific variation and environmental control. New Phytologist.

[b63] Ramel F, Birtic S, Cuiné S, Triantaphylidès C, Ravanat J-L, Havaux M (2012a). Chemical quenching of singlet oxygen by carotenoids in plants. Plant Physiology.

[b64] Ramel F, Birtic S, Ginies C, Soubigou-Taconnat L, Triantaphylidès C, Havaux M (2012b). Carotenoid oxidation products are stress signals that mediate gene responses to singlet oxygen in plants. Proceedings of the National Academy of Sciences of the United States of America.

[b65] Rossel JB, Wilson PB, Hussain D, Woo NS, Gordon MJ, Mewett OP, Howell KA, Whelan J, Kazan K, Pogson BJ (2007). Systemic and intracellular responses to photooxidative stress in *Arabidopsis*. The Plant Cell.

[b66] Schwartz SH, Qin X, Loewen M (2004). The biochemical characterization of two carotenoid cleavage enzymes from *Arabidopsis* indicates that a carotenoid-derived compound inhibits lateral branching. Journal of Biological Chemistry.

[b67] Tan BC, Schwartz SH, Zeevaart JAD, McCarty DR (1997). Genetic control of abscisic acid biosynthesis in maize. Proceedings of the National Academy of Sciences of the United States of America.

[b68] Terashima I, Hanba YT, Tazoe Y, Vyas P, Yano S (2006). Irradiance and phenotype: comparative eco-development of sun and shade leaves in relation to photosynthetic CO2 diffusion. Journal of Experimental Botany.

[b69] Thayer SS, Björkman O (1990). Leaf xanthophyll content and composition in sun and shade determined by HPLC. Photosynthesis Research.

[b70] Tian L, DellaPenna D, Zeevaart JAD (2004). Effect of hydroxylated carotenoid deficiency on ABA accumulation in *Arabidopsis*. Physiologia Plantarum.

[b71] Umehara M, Hanada A, Yoshida S (2008). Inhibition of shoot branching by new terpenoid plant hormones. Nature.

[b73] Watling JR, Robinson SA, Woodrow IE, Osmond CB (1997). Responses of rainforest understorey plants to excess light during sunflecks. Australian Journal of Plant Physiology.

[b74] Yamamoto HY, Bassi R, Ort DR, Yocum CF (1996). Carotenoids: localization and function. Oxygenic Photosynthesis: The Light Reactions.

[b75] Yazdanbakhsh N, Fisahn J (2010). Analysis of *Arabidopsis thaliana* root growth kinetics with high temporal and spatial resolution. Annals of Botany.

[b76] Yin Z-H, Johnson GN (2000). Photosynthetic acclimation of higher plants to growth in fluctuating light environments. Photosynthesis Research.

